# Phenolic Profiling of Albanian Honeys by LC–MS/MS: Gallic Acid as a Predictive Marker of Antioxidant Potential

**DOI:** 10.3390/molecules30204037

**Published:** 2025-10-10

**Authors:** Xhulieta Hamiti, Gjyliza Shallari, Blerina Pupuleku, Alp Yücel, Saffet Çelìk, Erhan Sulejmani, Pranvera Lazo

**Affiliations:** 1 Department of Chemistry, Faculty of Natural Sciences, University of Tirana, 1016 Tirana, Albania; pranvera.lazo@fshn.edu.al; 2 Department of Software Engineering, Faculty of Engineering, Canadian Institute of Technology, 1000 Tirana, Albania; gjyliza.shallari@cit.edu.al; 3 Department of Biology, Faculty of Natural Sciences, University of Elbasan “Aleksander Xhuvani”, 3001 Elbasan, Albania; blerina.pupuleku@uniel.edu.al; 4 Technology Research and Development Application and Research Centre, Trakya University, 22030 Edirne, Turkey; alpyucel@trakya.edu.tr (A.Y.); saffetcelik@trakya.edu.tr (S.Ç.); 5 Department of Food Technology, State University of Tetovo, 1200 Tetovo, North Macedonia

**Keywords:** honey, phenolic compounds, flavonoids, LC–MS/MS, gallic acid, antioxidant potential, Albania, mono-floral, poly-floral

## Abstract

Phenolic compounds are key contributors to the bioactivity and antioxidant potential of honey, yet reliable indicators for rapid quality assessment remain limited. This study characterized phenolic profiles in 44 mono-floral and poly-floral honey samples from 11 Albanian regions using LC–MS/MS method. Total phenolic content ranged from 29.8 to 171 mg·kg^−1^, with flavonoids accounting for 6.1–56.4% of total phenolics. Gallic acid was the dominant phenolic compound (5.5–127 mg·kg^−1^), which is strongly correlated with the total content of polyphenols (r = 0.863, *p* < 0.001). Analysis of variance (ANOVA) confirmed significant differences in phenolic and flavonoid levels across geographic zones and floral types, with mono-floral honeys consistently exhibiting higher bioactive parameters. These findings demonstrate that gallic acid may serve as a practical biochemical marker for estimating antioxidant potential in honey. This insight has implications for authenticity verification and quality control, particularly in regions like Albania where diverse floral sources contribute to honey variability. By linking phenolic composition to antioxidant potential, this study supports the broader application of phenolic markers for the standardization and valorization of honey as a functional food.

## 1. Introduction

Honey is a natural sweetener produced by bees and is recognized for its rich composition of phenolic acids and flavonoids, which largely determine its antioxidant potential and association with health benefits [1,2,3]. These compounds vary considerably according to floral source, geographic origin, and environmental factors [4,5,6]. Such variability directly influences honey’s bioactive potential and can serve as a basis for quality assessment and authentication of honey [7,8].

Phenolic acids (e.g., gallic, caffeic, chlorogenic acids) and flavonoids (e.g., quercetin, kaempferol, rutin) are frequently used as indicators of the antioxidant potential of honey [9,10,11,12]. Their content and composition are highly dependent on the nectar source. Melissopalynology (pollen analysis) combined with phenolic profiling is the standard approach to verify honey’s botanical origin [13], yet few studies have systematically evaluated the relationship between phenolic markers and antioxidant potential in honeys from under-studied regions.

Albania, situated in the Mediterranean and Southeastern European regions, is characterized by its rich biodiversity and diverse floral sources. Honey production has skyrocketed in recent decades [14], but data on the phenolic composition and antioxidant relevance of Albanian honeys remain scarce [15,16,17,18]. Previous studies have focused mainly on physicochemical parameters such as hydroxy-methyl-furfural (HMF) and diastase activity [19,20], while detailed LC–MS/MS phenolic profiling is still lacking.

Among phenolic compounds, gallic acid has been consistently reported as a major contributor to honey’s antioxidant potential. Reviews by Hadidi et al. (2024) and Olas (2020) have shown that gallic acid effectively neutralizes free radicals and reduces oxidative stress in vitro [21,22]. One recent study from Karlıdağ et al. (2025) further confirms significant correlations between gallic acid content and antioxidant assay results [23]. However, its suitability as a predictive marker for total phenolic content (TPC) and antioxidant potential has not yet been validated in Albanian honey varieties.

This study aimed to characterize the phenolic composition of 44 mono-floral and poly-floral Albanian honey samples using LC–MS/MS and to evaluate whether gallic acid can serve as a biochemical marker of antioxidant potential in honey. In addition, the phenolic profiles between mono-floral and poly-floral honey are different. Recent research has associated the phenolic content of honey with antioxidant, anti-inflammatory, and antimicrobial activities [24]. For example, Tlak Gajger et al. [25] found that phenolic acids such as caffeic and ferulic acids contribute significantly to antimicrobial activity (via membrane disruption and enzyme inactivation), even in honeys where hydrogen peroxide levels are low. Similarly, Bereksi-Reguig et al. [26] observed that among 37 Algerian honeys, those with higher TPC values expressed in GAE (acid gallic equivalents) (up to ~122 mg GAE/100 g) showed stronger activity against pathogenic bacteria. Review studies [27] reinforce that while phenolics are primary drivers of these bioactivities, other components (e.g., enzymes, organic acids, flavonoids) and some factors such as floral source and color also modulate the biological properties of honey.

## 2. Materials and Methods

### 2.1. Honey Samples

The samples were obtained from local beekeepers affiliated with the Beekeepers Association from August to September 2022. We collected 44 honey samples from 11 regions of Albania, with 4 samples from each region in total, which could be either mono-floral or poly-floral. The ratio of mono-floral to poly-floral samples varied across regions, depending on the practices of local beekeepers and the characteristic honey types of the areas from which the samples were collected (Table A1). The floral origin of each sample was determined through melissopalynological analysis, which identified the pollen types present in honey samples. Based on the obtained results, the samples were classified by plant source and geographic region, representing 11 distinct regions of the country.

### 2.2. Characterization of the Floral Origin of Honey

Melissopalynology was also used to verify the botanical origins declared by the producers. The pollen spectra of the 44 honey samples were determined using the method described by Louveaux et al. [28]. In all samples, the number of pollen grains exceeded 2300, fulfilling the quantitative criteria set by Von der Ohe et al. [9]. According to this classification, pollen types in honey are categorized into five frequency classes: D—predominant pollen: >45%; S—secondary (frequent) pollen: 16–45%; s—important minor pollen: 3–15%; r—rare pollen: 1–3%; i—incidental pollen: <1%. This classification provided a reliable basis for determining the floral type and confirming the mono-floral or poly-floral nature of honey.

### 2.3. Determination of Total Polyphenol Content (TPC) in Honey

The total phenolic content in honey was determined with slight modifications in the method adapted from Singleton et al. [29]. The acidified honey solution (200 μL) was diluted with 5 mL of deionized water followed by addition of 100 μL Folin–Ciocalteu reagent. After vortexing, 300 μL of 20% sodium carbonate solution was added to the mixture. The samples with 5 mL volume were then homogenized and allowed to stand at room temperature in the dark for 2 h. Absorbance measurements were recorded in triplicate at a wavelength of 765 nm using a SPECORD 250 PLUS UV-Vis spectrophotometer (Analytic Jena AG, Jena, Germany). The honey polyphenolic content was determined from a calibration curve made from gallic standards at the following contents (1, 15, 25, 35, 40, and 50 μg/mL). The TPC values are reported as gallic acid equivalents per gram of honey (mg GAE/100 g of honey, R^2^ = 0.9984).

### 2.4. Analysis of Phenolic Compounds

#### 2.4.1. Reagents

All chemical solvents and standards were of analytical grade. Standards were obtained from Sigma-Aldrich and Cayman Chemical (Sigma-Aldrich, Inc., St. Louis, MO, USA; Cayman Chemical Company, Ann Arbor, MI, USA); acetonitrile, methanol, acetic acid, and formic acid from Merck (Darmstadt, Germany). Standards stock solutions were prepared in methanol and diluted with extraction solvent (water/methanol/formic acid, *v*:*v*:*v*, 79:20:1). All stock solutions were stored at −20 °C. Further dilutions were carried out appropriately in extraction solvent.

#### 2.4.2. Extraction Methods

Method 1: Extraction was carried out by the modified methods of isolation of phenolic compounds developed by Fischer et al. 2011 [30]. An amount of 100 µL of sample was mixed with 900 µL of extraction solution (water/methanol/formic acid: *v*:*v*:*v*, 79:20:1). Afterward, samples were vortexed for 30 s. Then, the solution was homogenized using a sonicator (WiseClean, DAIHAN, Wonju-si, Republic of Korea) at 45 °C for 10 min, and centrifuged for 5 min at 13,500 rpm. The clear supernatant was injected into the LC–MS/MS system for quantitative analysis.

Method 2: An amount of 100 µL of sample was mixed with 200 μL of 2M HCl and vortexed for 30 s. Then, the solution was hydrolyzed using a sonicator at 90 °C for 40 min. After adding 700 µL of extraction solvent, samples were centrifuged for 5 min at 13,500 rpm and the clear supernatant was injected into the LC–MS/MS system for quantitative analysis. The reasons for using two different sample preparations: method 1: to analyze sugar-containing phenolic acids; method 2: to analyze basic phenolic acids. No filters were used in both methods because filters (PTFE, nylon, and cellulose acetate) are known to adsorb phenolic acids, especially luteolin, kaempferol, quercetin, and rutin, as reported by Bayram et al. 2020 [31].

#### 2.4.3. LC–MS/MS Conditions

LC was performed using an Agilent 6460 LC system (Agilent Technologies, Waldbronn, Germany). Chromatographic separation was carried out with a Agilent Zorbax SB-C8 column (150 × 3.0 mm, 3.5 μm particle size) and set at 35 °C with a mobile phase flow rate of 0.7 mL/min. Gradient elution mobile phases consisted of 5 mM ammonium acetate in water (solvent A) and acetic acid (% 0.1) in acetonitrile and methanol (*v*:*v*, 50:50) (solvent B). The gradient began initially at 96% A from 0 to 0.7 min, decreasing linearly to 2% at 2.3 min, this was maintained for 2.7 min, then reduced to 0% at 7.0 min before returning to initial conditions at 7.10 min. The total run time was 12 min. The autosampler was maintained at 4 °C and 10 μL of each sample was injected into the analytical column for compound analysis.

MS/MS analyses were accomplished on an Agilent LC–MS (Agilent Technologies, Waldbronn, Germany) 6460 triple quadruple mass spectrometer equipped with an electrospray ionization (ESI) interface. ESI was conducted in negative ion mode. The mass spectrometer was operated with a cycle time of 500 millisecond. To find the optimal parameters of ion path and ion source of the studied compound, quantitative optimization was performed by direct injection of standards using a HPLC Agilent 1260 (Santa Clara, CA, USA). Multiple reaction monitoring (MRM) mode of the dominant product ion for each solution was realized using the optimal conditions. The ion source parameters were as follows: Gas Temp: 300 °C; Gas Flow: 10 L/min; Nebulizer: 40 psi; Sheat Gas Heater: 400; Sheat Gas Flow: 10 L/min; Capillary: 3500 V. Samples analyzed with negative DMRM method (dynamic MRM). Dynamic MRM mode consists of comparison of pair ion (precursor and product ion *m*/*z* values) and LC retention times with standards served to confirm the identification of analyte in the samples. Ion pair was 152.9/107.9; 152.9/53.1 for 2,5-dihydroxybenzoic acid, 162.9/119; 162.9/92.8 for 2-hydroxycinnamic acid, 262.9/219.1; 262.9/153 for abscisic acid, 179/135.1; 179/117.3 for caffeic acid, 288.9/245; 288.9/205 for catechin and epicatechin, 352.9/191; 352.9/82 for chlorogenic acid, 197/169; 197/124 for ethyl gallate, 168.9/125; 168.9/78.8 for gallic acid, 345/239; 345/142.9 for gibberellic acid, 173.9/130.1; 173.9/128 for indole-3-acetic acid, 314.9/299.9; 314.9/151 for isorhamnetin, 209/164.9; 209/59.1 for jasmonic acid, 284.9/226.9; 284.9/93 for kaempferol, 284.9/150.9; 284.9/133 for luteolin, 317/178.8; 317/150.9 for myricetin, 579.1/458.9; 579.1/271 for naringin, 163.1/118.9; 163.1/93 for *p*-coumaric acid, 434.8/272.9; 434.8/167 for phlorizin, 211/124.1; 211/78 for propyl gallate, 153.1/109.1; 153.1/90.8 for protocatechuic acid, 301/178.9; 301/150.9 for quercetin, 226.9/184.9; 226.9/142.8 for resveratrol, 609/299.9; 609/270.9 for rutin, 136.8/93.1; 136.8/65 for salicylic acid, 222.9/208; 222.9/120.9 for sinapic acid, 196.9/182.1; 196.9/121.1 for syringic acid, and 193/177.9; 193/134.1 for trans-ferrulic acid. Data acquisition and processing were accomplished using MassHunter, the Agilent LC–MS software.

#### 2.4.4. Calibration Curve and Quantification

Phenolic acids concentrations in samples were calculated using the calibration curve that was prepared on the same day and analyzed in the same analytical run. All calibration curves were prepared following concentration: blank, 5, 10, 25, 50, and 100 ng/mL and each injected in triplicates. The linearity of all phenolic acids was R^2^ ≥ 0.995. These samples were analyzed according to the procedure described for sample preparation. LOD (limit of detection) and LOQ (limit of quantification) values of the phenolic acids (calculated using S/N ratio) are provided in Table A1 (Appendix A).

This analysis method has some important points. All phenolic acids were analyzed with a sensitivity down to 1 ng/mL and some of the isomer acids were separated under chromatography conditions. Validation parameters (LOD, LOQ, retention time, repeatability, and linearity) of selected phenolic compounds, flavonoids, and plant hormones analyzed by LC–MS/MS are presented in Table A2 (Appendix A).

### 2.5. Statistical Analysis

All samples were analyzed in triplicate, and the results are expressed as mean ± standard deviation (SD). Descriptive statistical analysis was performed to assess the levels, variation, sources, and the most dominant type of mono flower vs. poly flower honey. One-way and two-way ANOVA was conducted to determine whether statistically significant differences existed in phenolic compounds, total phenolics, and flavonoids across geographic zones and honey types (mono-floral vs. poly-floral). The significance of differences between means (*p* < 0.05) was assessed using Tukey’s test for the comparison of different regions honey samples, as outlined by Field [32]. Multivariate analysis (Pearson correlation and factor analysis, FA) was conducted to explore the associations and relationships among the studied physicochemical parameters and their dependence on chemical characteristics, weather conditions, sources and properties of bee feeding behavior, and regional factors. Pearson correlation coefficients were calculated for all phenolic compounds at a significance level of *p* < 0.05 to assess the strength and direction of linear relationships between variables. FA is a multivariate statistical technique that extracts a limited number of latent factors from linearly correlated variables by reducing big data to a few factors and providing insight into the factors that affected the distribution and origin of the parameters studied. Statistical analysis was performed using the software program MINITAB 21 (Minitab Statistical Software of the year 2021) [33].

## 3. Results and Discussion

### 3.1. Floral Profiling of Honey Samples

**Figure 1:**
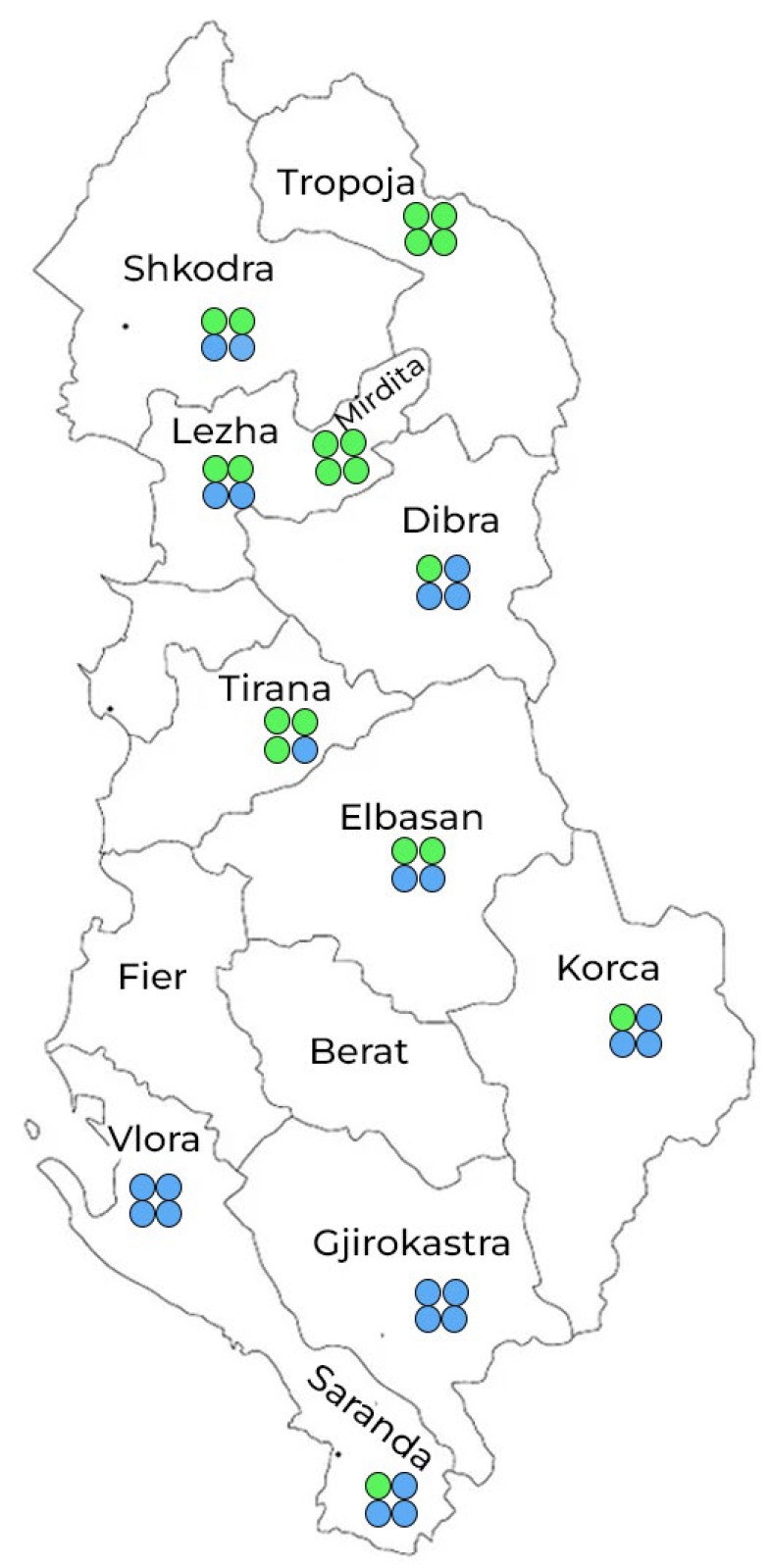
Sampling map of 44 honey samples from 11 Albanian regions (green = mono-floral honeys and blue = poly-floral honeys).

Forty-four honey samples were collected from 11 regions across Albania (MK1–MK11), representing a variety of ecological zones from the mountainous Tropoja (north) to the coastal Saranda (south) (Figure 1). Melissopalynology method [9,28] classified 20 samples as mono-floral and 24 as poly-floral. The dominant mono-floral types were *Castanea sativa* (chestnut, *n* = 10) and *Arbutus unedo* (*n* = 3). Poly-floral honeys typically included pollen from multiple botanical sources such as *Allium* spp., *Trifolium* spp., *Quercus*, *Cistus*, etc. In addition, some mono-floral samples showed pollen compositions corresponding to (S) mono-floral honey, such as the honey sample from Elbasan with 35% *Rosa* spp. pollen, indicated a significant but non-dominant botanical contribution. The types and quantities of pollen grains identified in the honey samples are presented in Table A3 (Appendix A).

### 3.2. TPC of Honey Samples by Region and Floral Types

**Figure 2:**
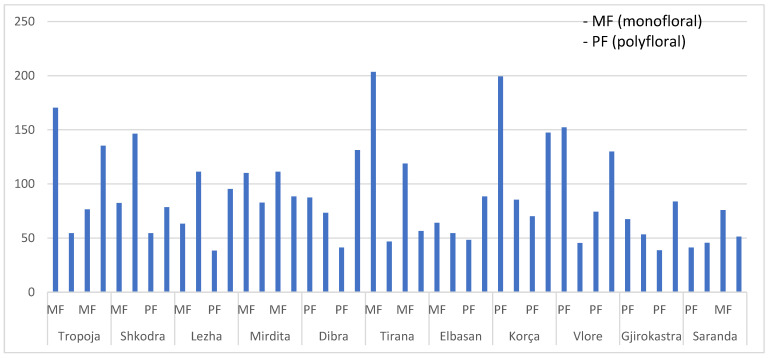
Total phenolic content (TPC, mg·kg^−1^) in honeys samples collected from 11 Albanian regions (20 mono-floral and 24 poly-floral).

In this study, the total phenolic content (TPC) was assessed for both mono-floral and poly-floral honey collected from 11 regions, comprising 44 samples in total. The results are summarized in Figure 2.

Total phenolic content (TPC) in Figure 2 ranged from 38 to 204 mg·kg^−1^ (with a mean of 88 mg·kg^−1^), indicating a pronounced diversity in overall phenolic richness. Higher TPC values were generally associated with mono-floral chestnut honeys from northern mountainous regions (~104 mg/kg), likely due to the phenolic-rich nectar of *Castanea sativa*. Mono-floral honeys from Tropoja, Shkodra, Mirdita, Tirana, and Korça have the highest TPC values, exceeding 100 mg·kg^−1^ in several cases. In contrast, poly-floral honeys from southern coastal zones tended to show lower TPC, often below 80 mg·kg^−1^, with the lowest levels in Saranda, Elbasan, and Gjirokastra, possibly influenced by differences in floral diversity, nectar composition, and climatic conditions [34].

One-way ANOVA indicated a significant effect of floral type on TPC (F_1_,_42_ = 4.59, *p* = 0.038). Mono-floral honeys (average value: 103 ± 41 mg·kg^−1^) exhibited significantly higher phenolic content compared to poly-floral honeys (average value: 76 ± 41 mg·kg^−1^), confirming that dominant nectar sources contributed to increased phenolic richness.

**Figure 3:**
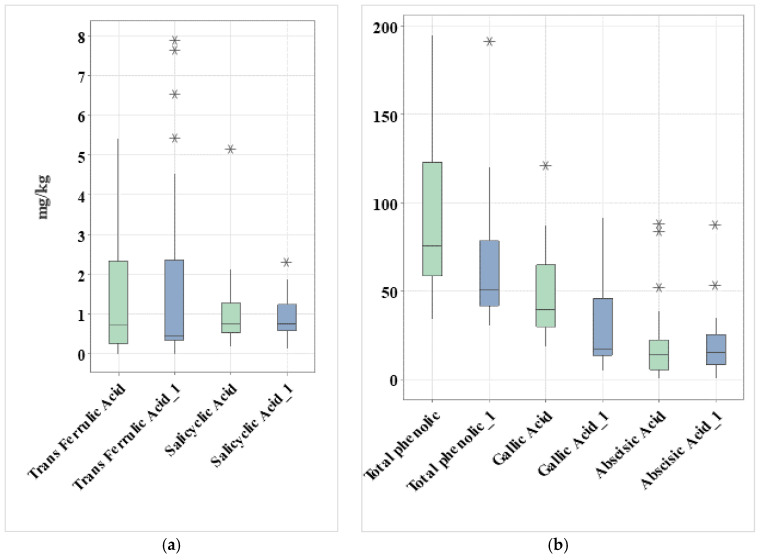
Boxplot (**a**) of trans-ferulic acid and salicylic acid contents (mg kg^−1^) in mono-floral (green boxes) honey samples and trans-ferulic acid_1 and salicylic acid_1 content (mg kg^−1^) in poly-floral (blue boxes) honey samples. Boxplot (**b**) of total phenols, gallic acid, and abscisic acid contents (mg kg^−1^) in mono-floral (green boxes) honey samples and total phenols_1, gallic acid_1, and abscisic acid_1 content (mg kg^−1^) in poly-floral (blue boxes) honey samples. Data analysis revealed several outliers, represented by points (*) outside the boxes. These values indicate unusually high or low measurements compared to the bulk of the dataset.

Boxplot (a) in Figure 3, showing selected phenolic acids (trans-ferulic acid and salicylic acid) in honey samples, indicates that trans-ferulic acid had higher median contents and broader variability compared to salicylic acid. Poly-floral samples (blue color) also showed greater variability, with some outliers exceeding 7 mg/kg, suggesting that specific floral sources are rich in this compound. By contrast, salicylic acid was consistently low in both groups (<2 mg/kg), with minimal variability. This indicates that while trans-ferulic acid may act as a distinguishing phenolic marker in mono-floral honeys, salicylic acid is more uniformly distributed and less discriminative between honey types.

Boxplot (b) in Figure 3, showing the total phenol content, gallic acid, and abscisic acid, showed the widest variation across samples. Gallic acid was consistently the predominant phenolic acid, while abscisic acid exhibited moderate levels with some high outliers, reflecting both floral and geographical influences. Total phenolic content (TPC) of mono-floral honeys had higher median values and wider dispersion (20–200 mg GAE/100 g), while poly-floral honeys clustered at lower values (~50–100 mg GAE/100 g). This pattern confirms the stronger phenolic richness of certain mono-floral honeys. Gallic acid seems present at moderate levels (10–70 mg/kg) in both groups but is slightly higher and more variable in mono-floral honeys. This suggests that gallic acid is a common constituent across honeys, but mono-floral types may accumulate more depending on botanical origin. Abscisic acid content was low overall (<30 mg/kg), but mono-floral honeys again showed greater variability, including several high outliers. This variability likely reflects the influence of specific nectar sources.

The presence of gallic acid across all 44 honey samples (up to 120 mg/kg) and its close clustering with total phenolic content (TPC) in factor analysis, indicate that it is a central component of the phenolic profile. This strong association highlights gallic acid as a major contributor to TPC and a potential biomarker for predicting the antioxidant activity of honey, consistent with previous reports linking gallic acid and TPC with antioxidant capacity in honey and plant-derived foods [2,6].

One study reported total phenolics ranging from 122 to 1170 mg GAE/kg in diverse European honeys [34,35]. Mono-floral Korça honey at approximately 148 mg/kg is representative, and its values falls in the mid-range compared to European benchmarks. Acacia honey from Lezha surpasses lighter honeys like acacia (~45 mg/kg) and aligns with average Czech samples (~110 mg/kg) [36].

Table 1 below summarizes the total phenolic content (TPC) of honey samples from different countries worldwide by botanical source, including comparative results for Albanian honey of selected botanical types [37].

Chestnut honeys stand out in Albania as the richest in phenolics, aligning with global trends where darker honeys are more phenolic-rich. Acacia and citrus honeys are low in phenolic content, consistent with their lighter color and nectar composition. Albanian poly-floral honeys appear significantly lower in phenolics compared to poly-floral honeys from Central and Western Europe, suggesting either different floral compositions or regional factors. These results confirm that botanical origin is the dominant factor shaping phenolic content in Albanian honeys, with regional chestnut honeys being especially promising for functional food applications.

### 3.3. Phenolic Compounds Content in Honey Samples

**Figure A1:**
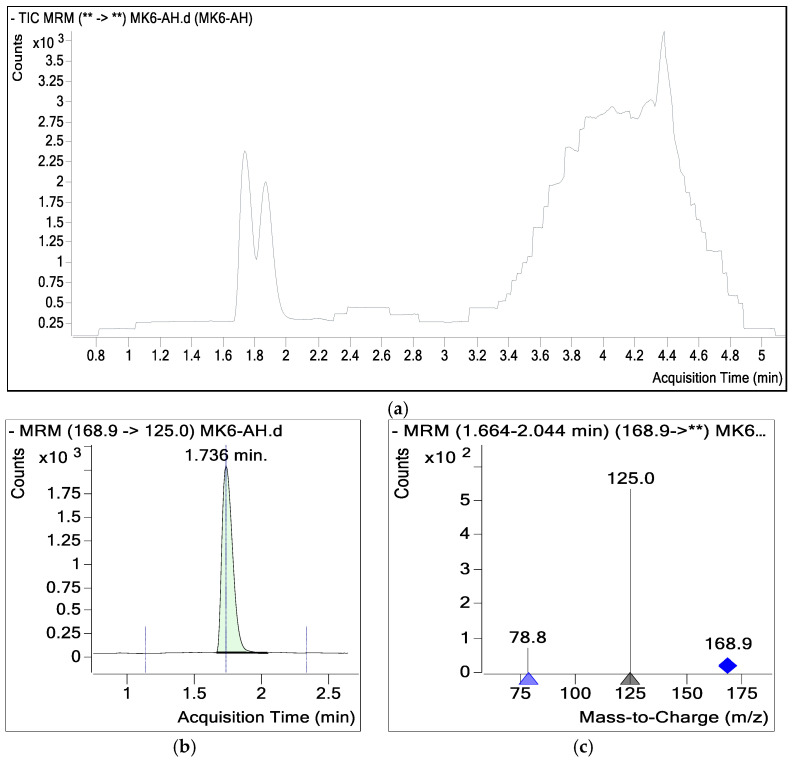
(**a**) Total ion chromatogram (TIC) obtained from LC–MS/MS analysis of honey sample H13 from Tirana, showing the elution of phenolic compounds between 3.0 and 5.0 min. (**b**) LC–MS/MS chromatogram showing MRM transition (*m*/*z* 168.9-125.0) for the gallic acid compound of honey sample H13 with the retention time of 1.736 min. The highlighted green peak represents the target analyte. (**c**) Product ion spectrum from LC–MS/MS analysis with characteristic fragment ions at *m*/*z* 125.0 and *m*/*z* 168.9, confirming the identity of gallic acid. Transitions marked with ** represent the primary ions used for quantitative evaluation, while unmarked transitions correspond to qualifier ions for compound confirmation.

LC–MS/MS equipment was used to identify the presence of polyphenolic compounds in all 44 honey samples from the studied geographical regions. The phenolic compounds were identified by matching their retention times with those of the available standards. Figure A1 shows a representative LC–MS/MS chromatogram TIC (total ion chromatogram) of H13 honey sample from Tirana (*Ericacea*), showing the elution of phenolic compounds between 3.0 and 5.0 min. The MS quantification results of this chromatogram are shown in Table A4 with corresponding retention times (RT, min) and final concentrations (ng/g). Major peaks were identified based on retention time and MS/MS fragmentation against authentic standards (e.g., gallic acid, caffeic acid, ferulic acid).

LC–MS/MS analysis identified 11 phenolic acids, 8 flavonoids, and 2 phenolic-like compounds (resveratrol and abscisic acid) across the samples. The phenolic profiles of the Albanian honey samples varied markedly across regions and botanical origins. Mono-floral samples (often Castanea-based) tend to show higher TPC and gallic acid levels. Poly-floral samples display more variability in flavonoids and sometimes higher levels of certain acids (e.g., caffeic acid). Some compounds (e.g., chlorogenic acid, resveratrol) are absent in several samples, indicating floral specificity.

Gallic acid was detected in all honey samples and in most cases, it occurs at relatively high concentrations, reaching values up to 120.99 mg kg^−1^, confirming it as the dominant phenolic compound in Albanian honeys. Its marked regional variability suggests strong botanical and geographic influences, supporting its potential use as a biomarker for antioxidant activity. The study shows that gallic acid is significantly higher in Greek oak honey [38], using it (with other phenolics) as an authenticity marker. This supports our statement about gallic acid’s dominance and regional/botanical variability as a potential biomarker of antioxidant activity.

Protocatechuic acid was present only in selected regions (3, 4, 9, and 11) at lower levels (≤19.1 mg kg^−1^), indicating flower-specific or seasonal production. Other phenolic acids, including 2,5-dihydroxybenzoic acid, caffeic acid, and chlorogenic acid, occurred at trace levels (<1 mg kg^−1^) but contributed to the overall antioxidant profile [39]. Samples with low polyphenol diversity but high levels of a dominant compound are likely to correspond to mono-floral honeys.

### 3.4. Chemometric Analysis of Phenolic Compounds in Honey Samples

Descriptive statistics analysis (Table 2) show that gallic acid was the predominant phenolic acid, with contents varying widely from 4.54 to 121 mg·kg^−1^ (mean = 39 mg·kg^−1^). This compound is often linked to strong antioxidant properties and is particularly abundant in chestnut honeys, which dominated the mono-floral category. Protocatechuic acid was the second most abundant (1.84–65 mg·kg^−1^; mean = 12 mg·kg^−1^), followed by smaller amounts of caffeic and p-coumaric acids, both recognized for their antimicrobial activity. Flavonoids, such as quercetin, kaempferol, and isorhamnetin, were consistently present across all samples, suggesting a broad botanical origin of these bioactive compounds. Abscisic acid displayed the greatest variability (0.350–88 mg·kg^−1^), potentially reflecting differences in plant physiology and environmental stress during nectar production. Flavonoid content was largely driven by quercetin, kaempferol, and isorhamnetin, with most other flavonoids detected at <1 mg·kg^−1^. This pronounced difference indicates that the antioxidant potential of these honeys is primarily determined by phenolic acids, reflecting the influence of dominant nectar sources such as *Castanea* and *Erica* [40]. Indeed, Di Marco et al. [40], in 2018 found that *Castanea* (chestnut) and *Erica* (heather) honeys are particularly rich in secondary metabolites and exhibit among the highest antioxidant activity compared to other mono-floral types in Italy.

The coefficient of variation (CV%) values in Table 2 for several phenolic compounds were high (up to 75% or more), indicating pronounced variability among samples. This reflects the influence of floral and geographical origin on the phenolic profile of honey, as well as possible differences in environmental and storage conditions. Such high CV% values are expected in honey studies; for example, Jaśkiewicz et al. 2025 [41] observed high intra-variety diversities in phenolic composition even within the same floral type, likely due to geographic origin, secondary nectar sources, and harvest time.

Statistical analysis supported these trends: two-way ANOVA revealed significant effects of both floral types (F = 49.3, *p* < 0.001) and geographic region (F = 4.9, *p* < 0.001) on TPC, while the interaction between floral type and region was not significant (*p* > 0.05). This suggests that floral origin and geographic factors independently shape the phenolic composition of Albanian honeys, highlighting the potential for phenolic profiling as a tool for both quality assessment and geographical authentication.

### 3.5. Multivariate Relationships Among Phenolic Compounds

Multivariate statistical tools such as factor analysis, are used to classify the honeys based on their phenolic compositions and geographical sources. Statistical analysis was performed using the MINITAB 21 software.

Pairwise Pearson correlations (−1 < r < +1) revealed strong and biologically relevant relationships between phenolic compounds in the Albanian honey samples. Total phenolic content exhibited a very strong positive correlation with gallic acid (r = 0.855, *p* < 0.001) and with abscisic acid (r = 0.725, *p* < 0.001), confirming gallic acid as the primary contributor to overall phenolic load. Significant positive associations were also observed between luteolin and 2,5-dihydroxybenzoic acid (r = 0., *p* < 0.001), and between isorhamnetin and salicylic acid (r = 0.568, *p* < 0.001), suggesting co-occurrence due to shared botanical origins. In contrast, several compounds displayed significant negative relationships, such as resveratrol with caffeic acetyl phenyl ester (r = −0.464, *p* < 0.001) and resveratrol with propyl gallate (r = −0.415, *p* < 0.01), indicating divergent patterns of occurrence (Table A5).

Principal component factor analysis (MINITAB 21 software) in Table 3 revealed four factors with eigenvalues > 1, jointly explaining 69.3% of the total variance in phenolic composition across honey samples and strong communalities with values above 0.7.

Factor 1 (20.4%) grouped luteolin, trans-ferulic acid, propyl gallate, caffeic acid, and 2,5-dihydroxybenzoic acid. These compounds often co-occur in nectar and pollen. Their strong communalities (>0.70 for most) indicate that these acids are well explained by the model and may represent a core phenolic group that contributes substantially to antioxidant activity in honey. Factor 2 (17.7%) was characterized by p-coumaric acid, quercetin, isorhamnetin, and ethyl gallate, reflecting the flavonoid profile often associated with poly-floral honeys. These compounds are strongly influenced by floral origin, particularly darker honeys such as chestnut or mixed poly-floral varieties. Their high loadings confirm that flavonoids act as distinct but complementary contributors to honey’s antioxidant potential. Factor 3 (17.7%) comprised caffeic acid phenyl ester, salicylic acid, and apigenin, suggesting the influence of propolis-derived phenolics and plant-specific metabolites. These compounds showed mixed positive and negative loadings, reflecting their uneven distribution across samples. Some are less common (e.g., caffeic acetyl phenyl ester, resveratrol) and may serve as secondary botanical markers. Their moderate communalities indicate that while they contribute to the factor structure, they capture more specific variations tied to floral or regional origin. Factor 4 (13.6%) was dominated by total phenolic content, gallic acid, and abscisic acid and clearly represents overall phenolic richness. The exceptionally high communality for TPC (0.958) indicates that this variable is almost fully explained by the factor solution. Gallic acid and abscisic acid also showed high loadings, confirming their role as strong indicators of honey quality and bioactivity.

Overall, the four-factor structure indicates that mono-floral honeys are more closely associated with distinctive phenolic acids and high total phenolic content (Factors 1 and 4), whereas poly-floral honeys are characterized by a diverse flavonoid profile (Factor 2). Factor 3 highlights the additional role of propolis-derived metabolites, contributing to the chemical complexity of the samples. These groupings indicate that both floral origin and secondary metabolite pathways significantly contribute to the phenolic signature of Albanian honeys.

**Figure 4:**
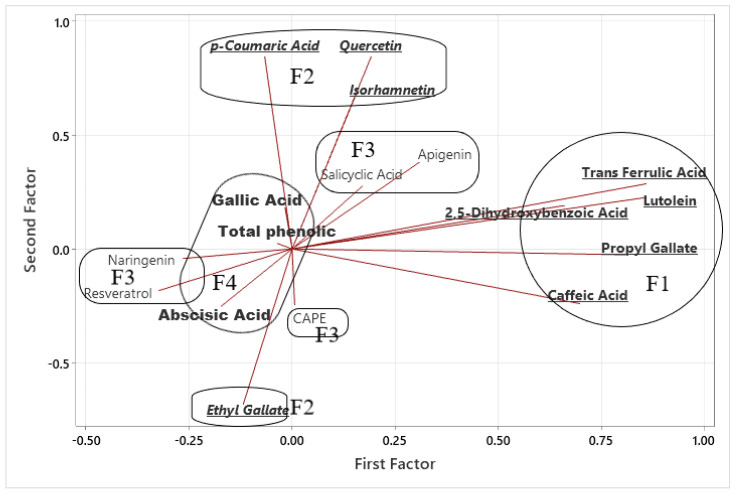
FA biplot distribution of honey samples under the principal components. The red lines represent the loadings of the phenolic compounds on the first two principal components. The direction of each line indicates the correlation between the compound and the components, while the length reflects the strength of the contribution. For example, trans-ferulic acid, luteolin, and propyl gallate showed strong positive loadings on Factor 1, whereas quercetin and p-coumaric acid contributed more strongly to Factor 2.

In Figure 4 of the FA biplot, principal component analysis delineated four distinct phenolic domains: core phenolic acids (F1), flavonoid-derived compounds (F2), propolis-derived metabolites (F3), and minor bioactives (F4). Such PCA-based discrimination aligns with Kędzierska-Matysek et al. [42], who used the PCA statistical analysis (a statistical methods equivalent to FA) to demonstrate that clustering of phenolic acids and flavonoids by FA is able to an effectively discriminate Polish honey varieties and correlate with antioxidant activity. Similarly, Nedić et al. [43] applied PCA to Serbian Tara Mountain honeys and found that specific phenolic compounds (e.g., p-coumaric, caffeic acids) underlie separation between mono-floral, poly-floral, and honeydew honeys—supporting our interpretation that F1–F4 reflect origin- and composition-dependent phenolic variability.

## 4. Conclusions

This study demonstrates that honey collected from 11 distinct regions of Albania is a valuable natural source of phenolic compounds, with gallic acid consistently identified as the predominant constituent. Significant variation in total phenolic content and individual phenolic compounds was observed across samples, with mono-floral honeys—particularly *Erica*-type—exhibiting the highest contents. A strong positive correlation between gallic acid and total amounts of phenolic content (r = 0.892, *p* < 0.001) supports the use of gallic acid as a reliable marker for estimating phenolic load and, by extension, antioxidant potential. These findings highlight the potential of phenolic profiling, and gallic acid in particular, for honey quality assessment and authenticity verification.

The strong association of gallic acid with TPC implies that variation in gallic acid levels may track much of the variation in TPC. Because phenolic content is often—and mechanistically—linked to antioxidant activity, gallic acid may serve as a biomarker or proxy for predicting antioxidant potential of honey.

Therefore, while our findings support gallic acid as a strong candidate biochemical marker, rather than a definitive predictor, we acknowledge the limitation that antioxidant efficacy cannot be attributed to gallic acid alone. Other phenolics, flavonoids, organic acids, and enzymes also play complementary roles.

The present analysis of phenolic compounds in honey was based on a one-year dataset. Consequently, longitudinal monitoring over multiple years will be essential to confirm these findings and provide a more comprehensive understanding of interannual variability.

## Figures and Tables

**Table 1:** Total phenolic contents (TPC in mg GAE *) in Albanian honeys according to botanical orgins compared with other countries.

Botanical Origin	Total Phenolic Contents mg GAE */100 g	Country/Region
*Robinia pseudoacacia* L.	6.32	Albania
	28.2–52.0	Croatia
*Chestnut* (*Castanea* spp.)	54.43–170.34	Albania/Tropoja
	82.65–111.21	Albania/Mirdita
	1.83	Korea
	0.12	Turkey
	487–1134	Portugal
	129.2–212.7	Croatia
*Citrus*	4.12	Albania
	14	Greece
	167.8	Italy
	83.85	India
Poly-floral	3.84–19.93	Albania
	236.94–1021.62	Poland
	744–1277	Portugal
	1199	Greece
	170	Mexico
	141	Poland
	81.22–983.04	Poland
	140.83	India/Shillong
	126.07	India/Mawsynram
	74.42	India Tezpur
	40.18–118.82	Argentine
	60.5	Algeria/Babors
	26.2–68.6	Estonia
	0.26	Turkey/Mesudiye

* GAE = Gallic Acid Equivalents.

**Table 2:** Descriptive statistics of phenolic compounds (mg·kg^−1^) identified in 44 Albanian honey samples by LC–MS/MS.

Phenolic Compounds	N	Mean	CV%	Min.	Q1	Median	Q3	Max.
Gallic Acid	44	38.29	71	4.54	16.77	30.2	53.94	121
Protocatechuic Acid	44	12.38	93	1.83	4.88	9.44	14.92	65.3
2,5-Dihydroxybenzoic	44	0.543	72	0.07	0.22	0.43	0.949	1.43
Caffeic Acid	44	0.589	115	0.05	0.21	0.36	0.672	3.72
Chlorogenic Acid	44	0.055	138	0.00	0.00	0.00	0.131	0.21
Salicylic Acid	44	1.020	82	0.13	0.55	0.76	1.242	5.16
Rutin	44	0.242	58	0.06	0.13	0.22	0.335	0.70
p-Coumaric Acid	44	0.331	138	0.00	0.00	0.14	0.501	1.55
Trans-Ferrulic Acid	44	1.620	137	0.00	0.33	0.54	2.337	7.90
Ethyl Gallate	44	0.010	96	0.00	0.01	0.01	0.012	0.03
Resveratrol	44	0.087	239	0.00	0.00	0.00	0.000	0.76
Propyl Gallate	44	0.002	78	0.00	0.01	0.00	0.038	0.01
Quercetin	44	0.327	83	0.03	0.09	0.21	0.561	0.97
Lutolein	44	0.111	77	0.01	0.05	0.09	0.167	0.33
Abscisic Acid	44	20.34	107	0.34	6.01	14.9	23.01	88.0
Naringenin	44	0.306	70	0.06	0.14	0.22	0.447	0.87
Genistein	44	0.064	147	0.01	0.01	0.02	0.075	0.33
Isorhamnetin	44	0.574	64	0.11	0.28	0.43	0.837	1.44
Kaempferol	44	0.660	68	0.17	0.28	0.55	0.948	1.88
Apigenin	44	0.040	64	0.00	0.02	0.04	0.059	0.09
Caffeic Acid Phenyl Ester	44	0.064	65	0.01	0.02	0.07	0.086	0.18
Total Phenolic Compounds	44	88.05	53	38.35	45.96	67.9	100.1	204

N—number of samples (44); Mean—average concentration of each phenolic compound; Min.—minimum concentration; Max.—maximum concentration. Q1 (first quartile)—the value below which 25% of the data fall; Q3 (Third quartile); Median—middle value of distribution. Note: A value of 0 indicates that compound was not detected.

**Table 3:** Factor loadings and communalities of phenolic compounds in Albanian honey samples.

Variable	F1	F2	F3	F4	Communality
Lutolein	0.856	0	0	0	0.787
Trans-Ferrulic Acid	0.854	0	0	0	0.822
Propyl Gallate	0.808	0	0	0	0.752
Caffeic Acid	0.697	0	0	0	0.573
2,5-Dihydroxybenzoic Acid	0.666	0	0	0	0.498
Quercetin	0	0.843	0	0	0.776
p-Coumaric Acid	0	0.843	0	0	0.731
Isorhamnetin	0	0.693	−0.485	0	0.758
Ethyl Gallate	0	−0.685	0	0	0.494
Naringenin	0	0	−0.88	0	0.871
Caffeic Acetyl Phenyl Ester	0	0	−0.878	0	0.833
Resveratrol	0	0	0.63	0	0.56
Salicylic Acid	0	0	−0.57	0	0.467
Apigenin	0	0	−0.565	0	0.559
Total phenolic	0	0	0	0.975	0.958
Gallic Acid	0	0	0	0.858	0.769
Abscisic Acid	0	0	0	0.677	0.579
Variance	3.461	3.013	3.007	2.307	11.787
% Variance	0.204	0.177	0.177	0.136	0.693

## Data Availability

Dataset available on request from the authors.

## Appendix A

**Table A1:** Limits of detection (LOD, µg/L) and quantification LOQ (µg/L) for phenolic acids, flavonoids and related compounds identified in Albanian honey samples by LC–MS/MS.

Compound	LOD (µg/L)	LOQ (µg/L)
2,5-Dihydroxybenzoic Acid	0.14	0.47
2-Hydroxycinnamic Acid	0.48	1.58
Abscisic Acid	0.15	0.49
Caffeic Acid	0.05	0.17
Catechin+Epicatechin	0.22	0.75
Chlorogenic Acid	0.09	0.29
Ethyl Gallate	0.09	0.28
Gallic Acid	0.31	1.02
Gibberellic Acid	0.95	3.16
Indole-3-acetic Acid	0.11	0.36
Isorhamnetin	0.06	0.18
Jasmonic Acid	0.06	0.19
Kaempferol	0.31	1.08
Luteolin	0.04	0.14
Myricetin	0.05	0.18
Naringin	0.07	0.24
*p*-Coumaric Acid	0.62	2.05
Phlorizin	0.02	0.08
Propyl Gallate	0.07	0.24
Protocatechuic Acid	0.04	0.15
Quercetin	1.01	3.35
Resveratrol	0.27	0.89
Rutin	0.04	0.13
Salicylic Acid	0.08	0.26
Sinapic Acid	0.91	2.97
Syringic Acid	1.01	3.34
Trans-Ferrulic Acid	0.11	0.35

**Table A2:** Validation parameters (LOD, LOQ, retention time, repeatability, and linearity) of some selected phenolic compounds, flavonoids, and plant hormones analyzed by LC–MS/MS.

Compound	LOD (µg/L)	LOQ (µg/L)	Ion Pair	RT *	% RSD ** (RT)	[M−H]^−^ *m*/*z*	R^2^ (Linearity)
Abscisic Acid	0.15	0.49	262.9/219.1; 262.9/153	4.338	3.6	[262.9]^−^	0.999
Jasmonic Acid	0.06	0.19	209.0/164.9; 209.0/59.1	4.423	1.7	[209.0]^−^	0.998
Naringenin	0.10	0.31	271.0/118.9; 271.0/150.9	4.370	0.9	[271.0]^−^	0.995
Apigenin	0.11	0.33	268.9/116.8; 268.9/150.7; 268.9/224.6	4.430	1.9	[268.9]^−^	0.999
Verbascoside	0.20	0.62	623.0/161.1; 623.0/461.2	3.952	2.1	[623.0]^−^	0.999
Hesperidin	0.27	0.83	609.0/301.0	4.062	3.9	[609.0]^−^	0.996
Indole-3-Acetic acid	0.22	0.68	173.9/128.0; 173.9/130.1	4.129	2.8	[173.9]^−^	0.998
Oleuropein	0.09	0.28	539.0/275.1; 539.0/307.2; 539.0/377.1	4.131	2.0	[539.0]^−^	0.995
Aloin A	0.05	0.17	417.0/297.1	4.207	3.4	[417.0]^−^	0.996

* RT—(Retention time, min.), ** RSD—Relative standard deviation.

**Table A3:** Botanical and geographical origin of honey.

Nr.	Region	No.	Honey Type	Melissopalynological Classification
1	Tropoja	H24	MF	Castanea 61%(D), Trifolium(r), Allium (i),
		H40	MF	Castanea 39%(S), Allium (r), Arbutus (i),
		H2	MF	Castanea 45%(D), Quercus(s), Thymus (i)
		H3	MF	Castanea 72%(D), Allium (s), Trifolium(r),
2	Shkodra	H6	MF	Castanea 54%(D), Cercis (r), Quercus(s)
		H7	MF	Castanea 67%(D), Cistus (r), Allium (i)
		H8	PF	Trifolium(r), Castanea (r), Mentha(r)
		H27	PF	Castanea (s), Trifolium(r), Cercis (r)
3	Lezha	H31	MF	Accacie 34%(S), Arbutus (i), Lavandula (r)
		H33	MF	Salvia 28%(S), Quercus (s), Mentha(r)
		H36	PF	Galega (r), Castanea (r), Quercus (r)
		H23	PF	Quercus (s), Punica (r), Castanea (i)
4	Mirdita	H5	MF	Erica 48%(D), Castanea(r), Trifolium(i)
		H35	MF	Castanea 31%(S), Allium (r), Castanea (i)
		H11	MF	Castanea 68%(D), Trifolium(r), Allium (i),
		H12	MF	Castanea 55%(D), Allium (s), Trifolium(r)
5	Dibra	H25	MF	Castanea 53% (D), Trifolium(r), Allium (i),
		H13	PF	Melilotus (s), Prunus (r), Juniperus (i)
		H14	PF	Allium (s), Trifolium(s), Castanea (r)
		H26	PF	Quercus (s), Punica (r), Castanea (i)
6	Tirana	H13	MF	Erica 58%(D), Arbutus (s), Cercis (r)
		H15	PF	Arbutus 59%(D), Helianthus (s), Castanea (i)
		H28	PF	Arbutus 63%(D), Allium (r), (S), Cercis (r)
		H29	PF	Mentha (s), Allium (r), Arbutus (i)
7	Elbasan	H39	MF	Arbutus 46%(D), Allium(r), Castanea (i)
		H32	MF	Rosa 35%(S), Castanea (s), Tordylium(s)
		H44	PF	Rubus (s), Pimpinella (r), Cercis (r)
		H15	PF	Melilotus(r), Trifolium(r), Castanea (r),
8	Korça	H16	MF	Staehelinauniflosculo 58%(D),Trifolium(r)
		H1	PF	Salvia (r), Medigago (r), Mentha(r)
		H4	PF	Trifolium(r), Castanea (r), Mentha(r)
		H19	PF	Quercus(s), Origanum (r), Trifolium(r)
9	Vlora	H30	PF	Ononis (s), Plantago (r), Arbutus (i)
		H37	PF	Robinia (r), Castanea (r), Mentha(r)
		H9	PF	Trifolium(r), Platanus(r), Mentha(r)
		H10	PF	Tamarix(s), Tordylium(s), Trifolium(r)
10	Gjirokastra	H34	PF	Trifolium(r), Platanus(r), Menth a(r)
		H43	PF	Thymus (r), Castanea (r), Helianthus (r)
		H17	PF	Medicago (r), Cercis (r), Mentha (r)
		H18	PF	Tamarix(s), Tordylium(s), Trifolium(r)
11	Saranda	H17	MF	Citrus (S), Trifolium (r), Lavandula
		H38	PF	Medicago (r), Cercis (r), Mentha (r)
		H41	PF	Castanea (r), Arbutus (i), Trifolium(r)
		H42	PF	Medicago (r), Cercis (r), Mentha (r)

H—sample code corresponding to each honey sample; (D)—mono-floral from one botanical source with dominant pollen >45%; (S)—mono-floral from one botanical source with dominant pollen 16–45%; (s), (i), (r)—pollen content in poly-floral honey (important minor, incident, rare);

**Table A4:** MS quantification results of LC–MS/MS chromatogram of H13 honey sample from Tirana.

Compound	RT (min)	Final Conc ng/g
Gallic Acid	1.736	120,990.80
Protocatechuic Acid	1.872	65,331.69
2.5-Dihydroxybenzoic Acid	2.183	974.35
Syringic Acid	3.586	nd
Caffeic Acid	3.714	200.09
Chlorogenic Acid	3.719	nd
Salicyclic Acid	3.783	437.51
Catechin	3.888	386.22
Verbascoside	4.062	nd
Gibbarellic Acid	4.157	nd
Hesperidin	3.944	nd
Rutin	4.037	204.48
2-Hydroxytranscinnamic Acid	4.019	nd
p-Coumaric Acid	4.053	nd
Naringin	4.012	nd
Trans Ferrulic Acid	4.246	654.98
Sinapic Acid	4.194	nd
Ethyl Gallate	4.085	3.49
Phlorizin	4.156	nd
Indole-3-Acetic Acid	4.221	nd
Oleuropein	4.164	nd
Myricetin	4.123	nd
Resveratrol	4.169	nd
Aloin A	4.139	nd
Propyl Gallate	4.220	nd
Quercetin	4.275	260.25
Lutolein	4.302	64.78
Abscisic Acid	4.337	445.78
Compound	RT	Final Conc.
Naringenin	4.387	85.75
Jasmonic Acid	4.389	nd
Genistein	4.370	69.18
Isorhamnetin	4.385	558.33
Kaempferol	4.386	354.79
Apigenin	4.421	28.99
Caffeic Acetyl Phenyl Ester	4.580	7.01

nd—not detected; RT—Retention Time; Final Conc.—Final Concentration.

**Table A5:** Pearson correlation coefficients (r).

Parameter 1	Parameter 2	Correlation	95% CI for ρ	*p*-Value
Total phenolic	Gallic Acid	0.855	(0.747, 0.918)	<0.001
Trans-Ferrulic Acid	2,5-Dihydroxybenzoic	0.414	(0.134, 0.633)	0.005
Propyl Gallate	2,5-Dihydroxybenzoic	0.483	(0.218, 0.682)	0.001
Lutolein	2,5-Dihydroxybenzoic	0.611	(0.381, 0.770)	<0.001
Trans-Ferrulic Acid	Caffeic Acid	0.548	(0.300, 0.727)	<0.001
Propyl Gallate	Caffeic Acid	0.519	(0.263, 0.707)	<0.001
Lutolein	Caffeic Acid	0.423	(0.141, 0.642)	0.005
Naringenin	Salicylic Acid	0.401	(0.118, 0.624)	0.007
Isorhamnetin	Salicylic Acid	0.568	(0.326, 0.740)	<0.001
Caffeic Acetyl Ester	Salicylic Acid	0.439	(0.163, 0.651)	0.003
Ethyl Gallate	p-Coumaric Acid	−0.461	(−0.666, −0.190)	0.002
Quercetin	p-Coumaric Acid	0.585	(0.349, 0.751)	<0.001
Isorhamnetin	p-Coumaric Acid	0.392	(0.108, 0.617)	0.008
Propyl Gallate	Trans-Ferrulic Acid	0.607	(0.378, 0.766)	<0.001
Lutolein	Trans-Ferrulic Acid	0.775	(0.618, 0.872)	<0.001
Quercetin	Ethyl Gallate	−0.522	(−0.709, −0.266)	<0.001
Naringenin	Resveratrol	−0.412	(−0.632, −0.132)	0.005
Apigenin	Resveratrol	−0.435	(−0.648, −0.158)	0.003
Caffeic Acetyl Ester	Resveratrol	−0.464	(−0.669, −0.194)	0.002
Propyl Gallate	Resveratrol	−0.415	(−0.634, −0.134)	0.005
Lutolein	Propyl Gallate	0.64	(0.420, 0.789)	<0.001
Apigenin	Lutolein	0.434	(0.154, 0.650)	0.004
Total phenolic	Abscisic Acid	0.725	(0.546, 0.841)	<0.001
Isorhamnetin	Naringenin	0.424	(0.146, 0.640)	0.004
Apigenin	Naringenin	0.401	(0.118, 0.624)	0.007
Caffeic Acetyl Ester	Naringenin	0.705	(0.516, 0.828)	<0.001
Apigenin	Isorhamnetin	0.516	(0.258, 0.705)	<0.001

95%. confidence interval (CI); *p*-values for pairwise associations.
